# CT-based radiomics models decode fibrosis content and molecular differences in pancreatic ductal adenocarcinoma: a multi-institutional study

**DOI:** 10.1186/s13244-025-02036-z

**Published:** 2025-09-12

**Authors:** Fangqing Wang, Yang Sun, Jianwei Xu, Yufan Chen, Hui Zhang, Guotao Yin, Dexin Yu

**Affiliations:** 1 Department of Radiology, Qilu Hospital, Shandong University, Jinan, China; 2 Department of Radiology, Liaocheng Traditional Chinese Medicine Hospital, Liaocheng, China; 3 Department of Surgery, Qilu Hospital, Shandong University, Jinan, China; 4 Department of Radiology, Shandong Provincial Hospital Affiliated to Shandong First Medical University, Jinan, China; 5 Department of Pathology, Qilu Hospital, Shandong University, Jinan, China

**Keywords:** CT-based radiomics model, Fibrosis content, Molecular differences, Prognosis, Pancreatic ductal adenocarcinoma

## Abstract

**Objectives:**

To develop a CT radiomics model for predicting fibrosis grade in pancreatic ductal adenocarcinoma (PDAC) and to investigate the underlying prognosis value and biological basis.

**Methods:**

Patients with resected PDAC were retrospectively included from three institutions. Evaluating tumor fibrosis content using fibrotic pixels proportion through Masson staining of postoperative pathological sections. Radiomics features from preoperative contrast-enhanced CT (CECT) were extracted and used to develop models in the training cohort. The diagnosis performance was further validated in the two test cohorts. The outcome cohort, including patients with advanced PDAC undergoing neoadjuvant chemotherapy, was used to evaluate the predictive value of the model for overall survival (OS) and disease-free survival (DFS), which were investigated using the Kaplan–Meier method and log-rank test. RNA sequencing data from a prospective biological basis cohort were conducted to explore the biological processes underlying the radiomics model.

**Results:**

Among 215 patients (median age 60.89 years, 142 men) used for radiomics modeling, 132 (61.40%) were confirmed as high fibrosis content. The combined phase (CP) radiomics model, which included all CECT radiomics features, showed the best performance for predicting fibrosis grade, with AUCs of 0.831, 0.785, and 0.746 in training, internal test, and external test cohorts. OS (*p* = 0.011) and DFS (*p* = 0.022) can be categorized using the CP radiomics model in the outcome cohort. RNA-seq indicated that different CP models were associated with fibrotic production and remodeling processes.

**Conclusion:**

The CP radiomics model showed the best performance in predicting fibrosis grades in PDAC.

**Critical relevance statement:**

Fibrosis grading is of prognostic and neoadjuvant chemotherapy efficacy evaluation significance, and the CT-based combined phase radiomics model established in our study will facilitate risk stratification and selection of personalized treatment strategies for patients. Furthermore, underlying biological processes demonstrated in the radiomics model will offer valuable insights into their interpretability and clinical translation.

**Key Points:**

- Fibrosis grading is of prognostic significance in pancreatic ductal adenocarcinoma (PDAC), but lacks a reliable preoperative assessment.
- The CT-based combined phase (CP) radiomics model predicts fibrosis grading effectively in PDAC.
- The CP radiomics model demonstrated prognostic and neoadjuvant chemotherapy efficacy evaluation value and underlying biological processes, which related fibrotic production and remodeling processes.

**Graphical Abstract:**

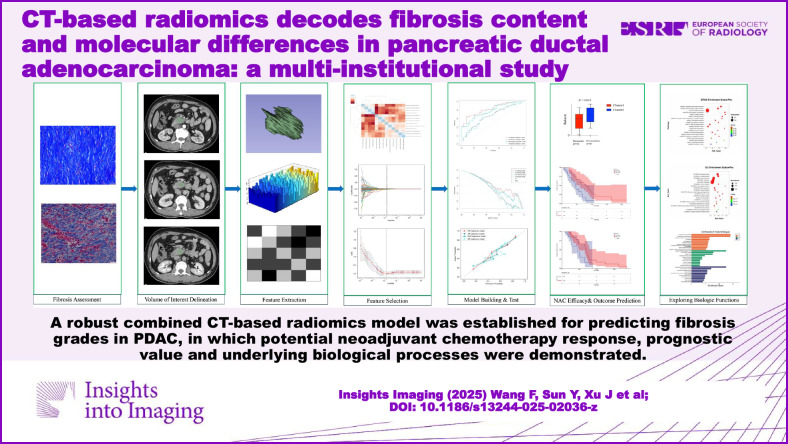

## Introduction

Pancreatic ductal adenocarcinoma (PDAC) is the leading primary pancreatic malignancy with a 5-year survival rate less than 12% [1, 2]. Surgery remains the only curative treatment for PDAC. As the majority of patients are diagnosed at an advanced stage, less than 20% patients can be completely resection [3]. Chemotherapy is recommended for patients with locally advanced PDAC, but provides only limited improvement. Abundant tumor matrix components are associated with chemotherapy failure [4]. Fibrosis forms a dense physical barrier around the tumor, hindering tumor vascularization and impairing drug delivery to cancer cells [5, 6]. Nab-paclitaxel has the unique capacity to reduce cancer-associated fibroblasts and to reduce tumor stroma [7]. Locally advanced PDAC patients with a high tumor stroma ratio have benefitted from nab-paclitaxel and gemcitabine but not from other gemcitabine-based regimens [8]. Furthermore, replacing tumor cells with fibrous tissue is one of the main pathological indicators for evaluating the efficacy of neoadjuvant chemotherapy (NAC) from no reaction to complete remission [9]. Thus, accurately assessing fibrosis is crucial for monitoring therapeutic effects and treatment planning.

Noninvasive CT imaging is crucial for the diagnosis and evaluation of PDAC. Previous studies have demonstrated that various radiologic characteristics, such as tumor diameter, peripancreatic tumor infiltration, denoting attenuation differences between tumor and surrounding pancreas, are independent predictors of PDAC fibrosis content [10, 11]. Even so, the evaluation of imaging features is highly subjective and can lead to discordance among radiologists. Radiomics, a recently developed technique, can help predict tumor phenotype and heterogeneity and provide information about tumor biological behaviors based on a large amount of high-throughput data [12, 13]. Currently, CT-based radiomics has been widely used to stage hepatic fibrosis and has shown considerable predictive value [14, 15]. Hepatic fibrosis and pancreatic fibrosis exhibit similar histopathological changes and share pathways involving activated stellate cells [16]. However, to our knowledge, studies of PDAC fibrosis based on CT radiomics are currently lacking in the literature.

Additionally, increasing evidence has shown the close association between radiomic features and specific biological pathways in tumors [13, 17, 18]. Therefore, further research is required to support the robustness of the radiomics features for fibrosis prediction and to explore the specific molecular mechanisms, providing biological validation and promoting clinical translation [19].

Thus, the purpose of our study was to construct a radiomics model based on preoperative contrast-enhanced computed tomography CT (CECT) imaging for predicting different fibrosis content, test the model in internal and external test cohorts, and to understand the biologic functions associated by analyzing RNA sequencing (RNA-seq) data.

## Methods

### Study patients

**Fig. 1:**
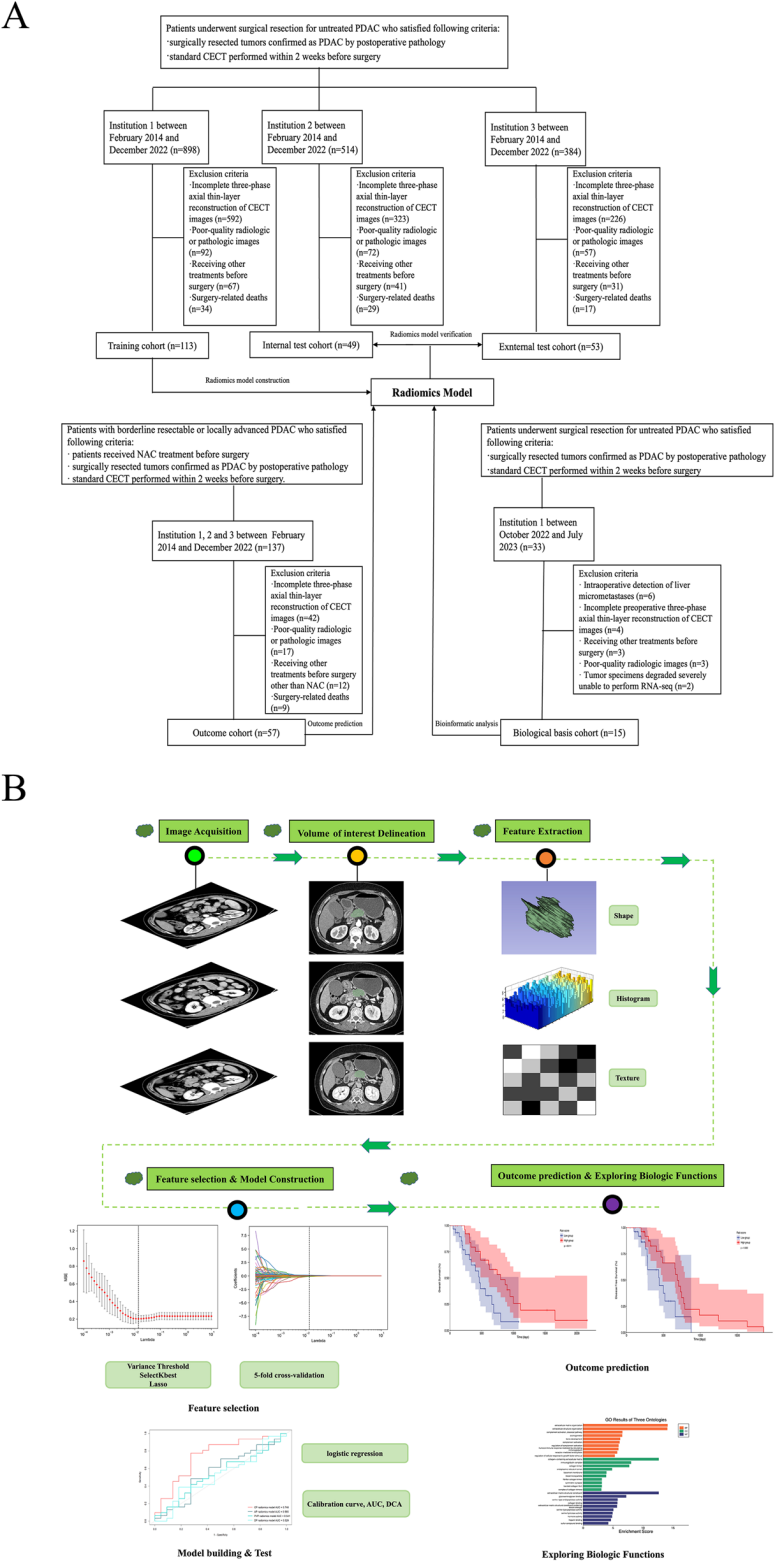
Patient selection and study design. **A** Flow diagram of the patient selection process for the training cohort, internal test cohort, external test cohort, outcome cohort and biological basis cohort from three medical centers. **B** Workflow for radiomics feature extraction, model building, and downstream analysis. Lasso, least absolute shrinkage and selection operator; AUC, area under the curve; DCA, decision curve analysis; RNA-seq, RNA sequencing

Patients from three separate institutions who underwent surgical resection for PDAC between February 2014 and December 2022 were retrospectively collected for radiomics model construction and verification: a training cohort from insititution 1; an internal test cohort from insititution 2, and an external test cohort from insititution 3. The outcome cohort included patients who underwent surgical resection after NAC between February 2014 and December 2022 from three institutions, to assess the efficacy of NAC and the prognostic value of the radiomics model. The prospective biological basis cohort comprised patients who underwent surgical resection for PDAC at institution 1 between October 2022 and July 2023. Fresh tumor specimens were obtained and used for RNA-seq analysis, which was performed on the Illumina Novaseq XP platform. The inclusion and exclusion criteria, along with detailed NAC regimens for patients, are explained in Appendix E1–E4. The detailed patient recruitment process is shown in Fig. 1A. The study was approved by the Institutional 1 Ethics Review Board (KYLL-202111-222). The requirement for written informed consent was waived for the retrospective data cohorts (2021253). Written informed consent was obtained from each prospectively enrolled patient. The study adhered to the CheckList for EvaluAtion of Radiomics research (Supplementary 1) and and METhodological RadiomICs Score (Supplementary 2).

### CT acquisition and data collection

All patients underwent abdominal three-phase CECT-arterial phase (AP), portal venous phase (PVP), and delayed phase (DP) within 2 weeks before surgery. Detailed patient preparation, imaging scheme, and acquisition parameters are summarized in Appendix E5. Two radiologists with 7 and 10 years of abdominal experience independently reviewed the CT imaging features. Radiological features were selected from the variables in the PDAC radiology reporting template recommended by the Society of Abdominal Radiology and the American Pancreatic Association [20]. The features are detailed in Appendix E6.

### Clinicopathological data evaluation

Relevant clinical and conventional pathological data of PDAC patients were retrieved from medical records. The histopathological response for PDAC following NAC was assessed using the tumor regression grading (TRG) system recommended by the College of American Pathologists [21]. Finally, patients were divided into remission and nonremission groups. Detailed information on clinicopathological variables and TRG grading system are presented in Appendix E7.

### Fibrosis content assessment

**Fig. 2:**
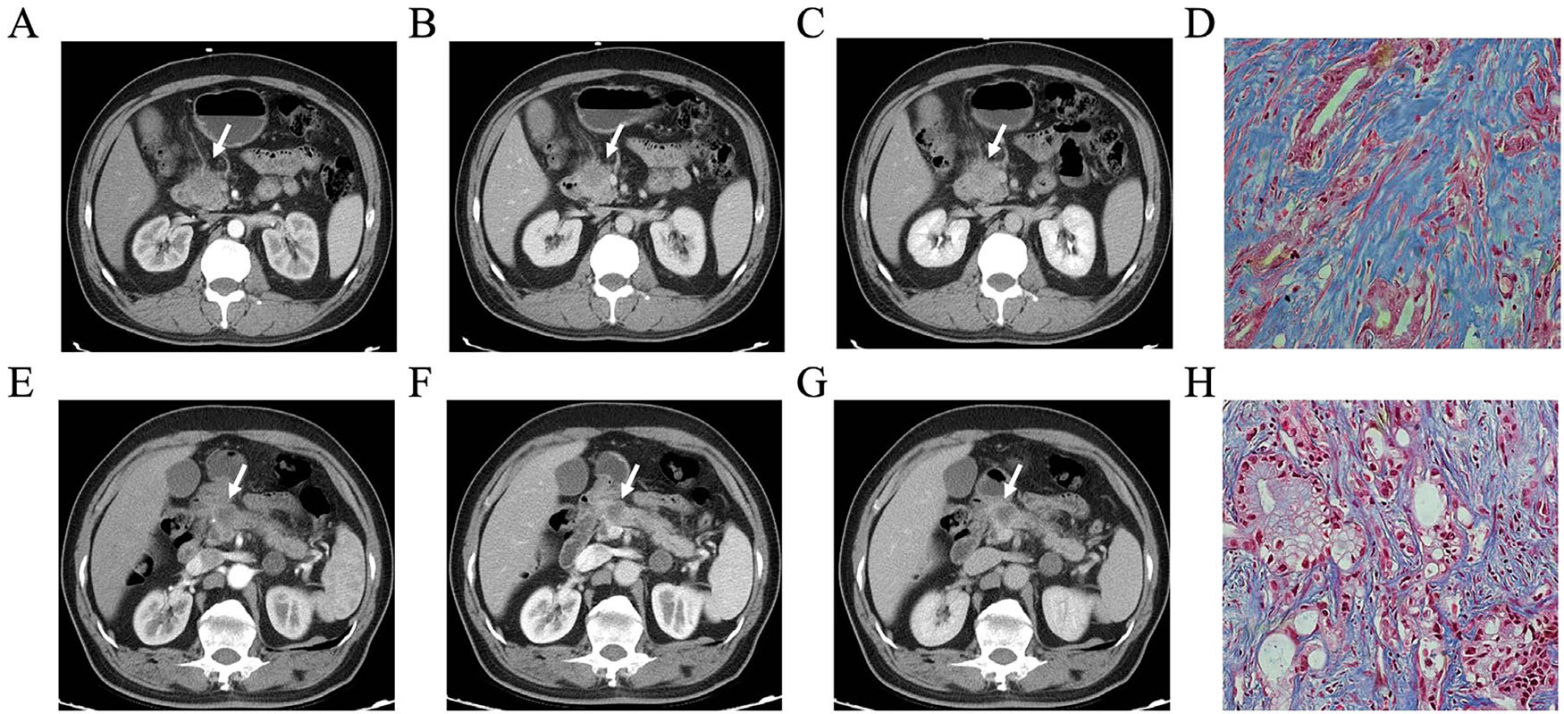
A 48-year-old female was diagnosed with PDAC, and CT images demonstrated that the tumor diameter was 27 mm with peripancreatic tumor infiltration (**A**–**C**), and the CA19-9 level was 254.86 U/mL (**D**). The tumor showed a high fibrosis content of 62.35% (Masson stain; original magnification, × 400, blue represents fibrosis). A 59-year-old man was diagnosed with PDAC, and CT images demonstrated that the tumor diameter was 21 mm without peripancreatic tumor infiltration (**E**–**G**), the CA19-9 level was 429.74 U/mL. **H** The tumor showed a low fibrosis content of 23.53%. (Masson stain; original magnification, × 400, blue represent fibrosis)

Pathology slides of the postoperative tumor specimens were reviewed by two pancreatic pathologists with 7 and 10 years of experience, respectively, and evaluated fibrosis content independently by Masson staining. The measurement is detailed in Appendix E8. Fibrosis content was graded as follows [22]: Grade 1, 0–25%; Grade 2, 26–50%; Grade 3, 51–75%; Grade 4, 76–100%. These were further classified into two groups: low fibrosis group (Grade 1 and Grade 2) and high-fibrosis group (Grade 3 and Grade 4). Masson staining of PDAC was shown in Fig. 2.

### Disease-free survival (DFS) and overall survival (OS)

The patients were followed up at 1, 3, 6, and 9 months postoperatively and every 3–6 months thereafter. All follow-up examinations included carbohydrate antigen 19-9 (CA19-9) measurements and imaging (CECT, contrast-enhanced MRI, ultrasonography, or positron emission tomography). DFS was defined as the time from the surgery to disease recurrence or death, whichever occurred first. OS was defined as the time interval between the surgery and the date of death of any cause, or the last follow-up visit or the study end date of December 31, 2023.

### Image segmentation and radiomic feature extraction

The workflow of the radiomics is shown in Fig. 1B. Two radiologists with 7 and 10 years of abdominal experience performed tumor segmentation and radiomics feature extraction by using 3D Slicer-based open-source software (Version 4.8.1, www.slicer.org). The details are provided in Appendix E9. Subsequently, 1329 radiomic features were extracted from AP, PVP and DP phases of segmentation, respectively, giving a total of 3987 features for the combined phase (CP) radiomics features. The intraclass correlation coefficients (ICCs) were calculated to assess intra- and inter-observer reproducibility of the extracted radiomic features; ICC > 0.75 were selected as robust radiomic features for further analysis.

### Unsupervised clustering

Unsupervised agglomerative hierarchical or k-means clustering of robust features was performed to identify groups of patients with similar radiomic feature patterns in the training cohort. Clusterability was evaluated by Hopkins statistic H. The optimal number of clusters was determined by average silhouette statistic. Stability of the resulting clusters was assessed by Jaccard bootstrapping.

### Model building and testing

The variance threshold, selectKbest and least absolute shrinkage and selection operator (Lasso) methods are sequentially used to gradually obtain the optimal features from AP, PVP, DP, and CP images in the training cohort. Subsequently, the radiomic scores (Rad-score) of each period were calculated from the optimal radiomic features and their respective coefficients. Uni- and multivariate logistic regression analyses were used to develop the clinical-radiomics fusion model in the training cohort. Prediction performance was assessed with receiver operating characteristic (ROC) curve analysis and parameters including area under the curve (AUC), sensitivity (SEN), specificity (SPE) and accuracy (ACC). Hosmer–Lemeshow goodness-of-fit test was performed, and calibration curves were then generated. Decision curve analysis (DCA) was used to evaluate the clinical practicability. Furthermore, patients in the training, internal and external cohorts were divided into high and low groups based on the 0.5 value of the Rad-score. The Kaplan–Meier method was used to generate survival curves, and the log-rank test was performed to compare OS and DFS between the two groups.

### Outcome prediction and exploring biologic functions

Patients were divided into high and low groups based on the 0.5 value of the CP Rad-score in the outcome cohort. The Kaplan–Meier method was used to generate survival curves, and the log-rank test was performed to compare OS and DFS. Student’s *t*-test was used to compare the Rad-score between the remission and nonremission groups. Biological basis cohort patients were divided into low- and high CP Rad-score groups to determine the potential biological changes associated with the radiomics features. Transcriptomic analysis of the RNA-seq data is explained in Appendix E10.

### Statistical analysis

Statistical analyses were performed using SPSS (version 22.0) and R software (version 3.6.1). Student’s *t*-test, Mann–Whitney U test and Kruskal–Wallis H test were used to assess the differences of continuous variables. Chi-square test and Fisher’s exact test were used to assess the differences of categorical variables. The power analysis was performed by using the pwr.t2n.test, and the empirical effect size was also calculated. *p* < 0.05 was defined as significant. Estimation of sample size for radiomics model construction and the R software packages used are described in Appendix E11, E12.

## Results

### Patient characteristics

A total of 215 patients (age 60.78 ± 10.06 years, range 28–84 years) were included. The clinical, radiological and pathological characteristics of patients in the training cohort, internal and external test cohorts are summarized in Table 1. No significant difference in all characteristics (*p* > 0.05) between the three cohorts. The characteristics of patients in the internal and external test cohort between low- and high-fibrosis groups were observed in Table S1.

**Table 1:** Baseline patient characteristics

Characteristics	Training cohort (*n* = 113)	Internal test cohort (*n* = 49)	External test cohort (*n* = 53)	*p*-value
Patient demographics				
Gender (male)	79 (69.91)	34 (69.39)	29 (54.72)	0.133
Age (years)*	60.77 ± 9.40	59.51 ± 11.78	62.13 ± 9.70	0.446
BMI (kg/m^2^)*	23.10 ± 2.93	23.14 ± 3.40	23.67 ± 3.55	0.596
Clinical parameters				
Hypertension (yes)	29 (25.66)	18 (36.73)	21 (39.62)	0.134
Diabetes (yes)	25 (22.12)	12 (24.49)	17 (32.08)	0.384
Smoking (yes)	48 (42.48)	24 (48.98)	16 (30.19)	0.138
Drinking (yes)	52 (46.02)	23 (46.94)	15 (28.30)	0.070
Lymphocyte ratio (%)*	27.62 ± 10.11	24.74 ± 7.99	28.57 ± 9.08	0.114
Monocyte ratio (%)*	7.51 ± 2.48	7.52 ± 2.92	7.59 ± 2.11	0.986
Neutrophil ratio (%)*	62.54 ± 9.90	64.91 ± 9.64	60.50 ± 9.35	0.103
Albumin (g/L)*	41.79 ± 5.28	41.62 ± 6.25	40.32 ± 6.39	0.362
Globulin (g/L)*	25.80 ± 4.70	26.23 ± 4.72	24.58 ± 4.97	0.231
Albumin and globulin ratio (%)*	1.66 ± 0.36	1.61 ± 0.33	1.67 ± 0.29	0.655
CA19-9 (U/mL)*	395.94 ± 363.67	316.68 ± 340.73	416.21 ± 352.27	0.344
CA12-5 (U/mL)*	34.06 ± 51.32	21.47 ± 21.19	20.50 ± 16.26	0.089
CEA (ng/mL)*	5.00 ± 5.63	5.58 ± 7.67	5.63 ± 7.84	0.826
Radiologic features				
Location (head)	66 (58.41)	28 (57.14)	33 (62.26)	0.852
Tumor necrosis (yes)	64 (56.64)	28 (57.14)	25 (47.17)	0.474
Calcification (yes)	5 (4.42)	1 (2.04)	3 (5.66)	0.649
Peripancreatic tumor infiltration (yes)	106 (93.81)	44 (89.80)	47 (88.68)	0.469
Distal main pancreatic duct dilation (yes)	94 (83.19)	39 (79.59)	44 (83.02)	0.849
Adjacent pancreatic parenchymal atrophy (yes)	45 (39.82)	20 (40.82)	23 (43.40)	0.909
Arterial involvement (yes)	76 (67.26)	32 (65.31)	36 (67.92)	0.957
Venous involvement (yes)	78 (69.03)	30 (61.22)	31 (58.49)	0.354
Pathological indicators				
Maximal diameter (cm)*	3.91 ± 1.66	3.97 ± 1.72	3.36 ± 1.49	0.095
Perineural invasion (yes)	88 (77.88)	31 (63.27)	39 (73.58)	0.154
Microvascular invasion (yes)	32 (28.32)	14 (28.57)	19 (35.85)	0.591
T stage (T1-T2)	71 (62.83)	30 (61.22)	39 (73.58)	0.323
N stage (N0)	75 (66.37)	34 (69.39)	37 (69.81)	0.878
Differentiation (poor)	41 (36.28)	15 (30.61)	21 (39.62)	0.631
Fibrosis content (%)*	56.40 ± 19.46	52.52 ± 22.76	49.58 ± 14.80	0.093

Unless indicated otherwise, data are number of patients, and data in parentheses are percentages

*BMI* body mass index, *CA19-9* carbohydrate antigen 19-9, *CA12-5* carbohydrate antigen 12-5, *CEA* carcinoembryonic antigen

* Data are means ± SDs

### Radiomics features capture the clinical, radiological, pathological and prognostic differences among patients with PDAC

**Fig. 3:**
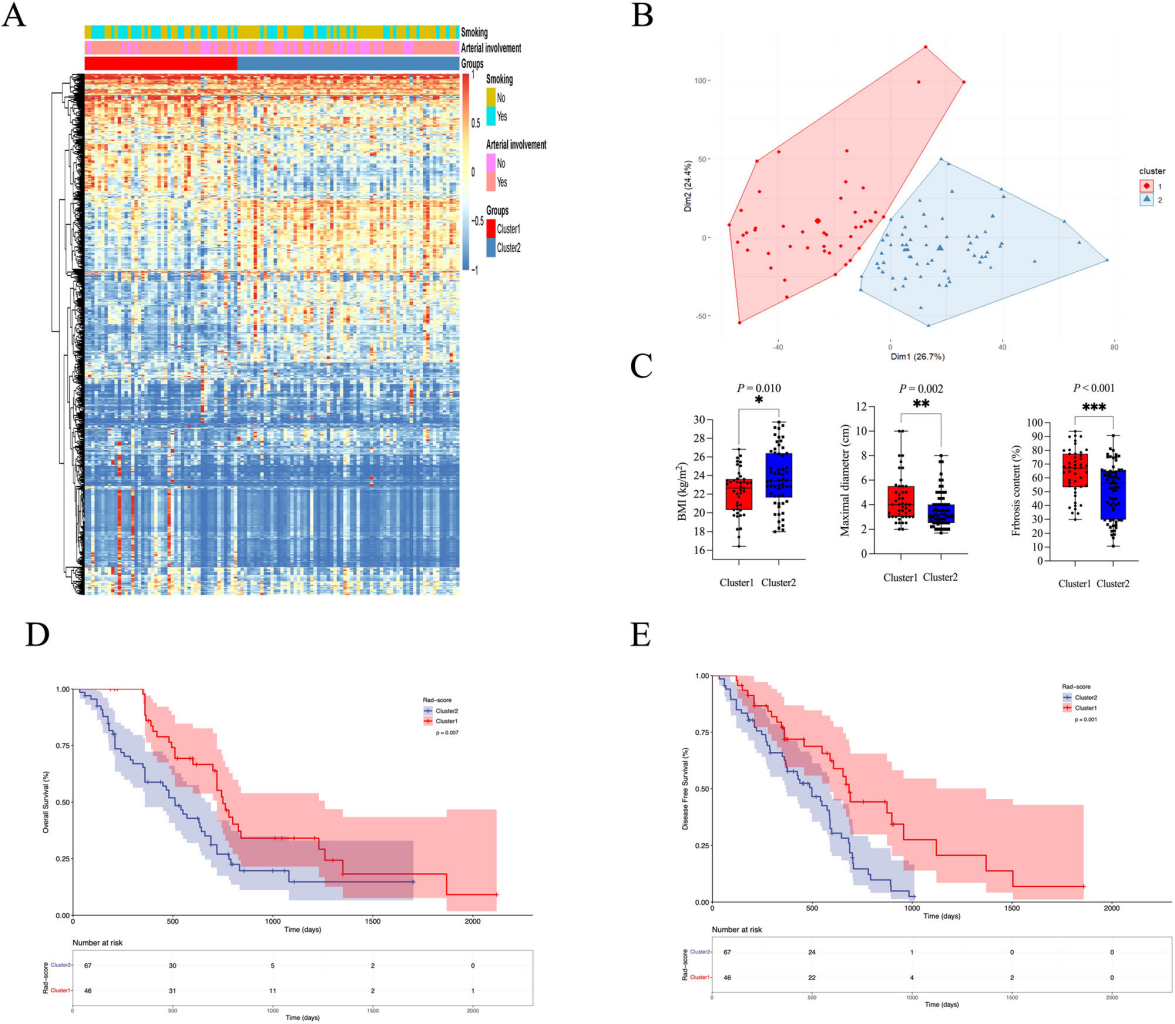
Unsupervised k-means clustering of radiomic data from PDAC patients. **A** Heatmap summarizing the k-means clustering results (training cohort, *n* = 113). Associations between two identified radiomic patient clusters with categorical parameters with significantly different (top) are shown. **B** k-means cluster plot indicating two stable clusters (cluster 1, red and cluster 2, blue). **C** Box plots show a significant difference in categorical clinical parameters of BMI, maximal diameter, and fibrosis content between cluster 1 and cluster 2. **D** Kaplan–Meier curves for OS defined as time to all-cause death. **E** Kaplan–Meier curves for DFS defined as time to first tumor recurrence or metastasis

Unsupervised clustering of the 3061 robust radiomics features revealed two distinct and stable patient clusters exhibiting different radiomics tumor profiles (Fig. 3A, B). Subanalysis by k-means clustering confirmed their statistical stability (Jaccard coefficients > 0.90). Patients in cluster 1 had a higher frequency of smoking (*p* = 0.034) and arterial involvement (*p* = 0.039) than those in cluster 2 (Fig. 3A and Table S2). Moreover, compared to patients in cluster 2, cluster 1 had a higher fibrosis content (*p* < 0.001), smaller tumor maximal diameter (*p* = 0.002) and lower BMI (*p* = 0.010) (Fig. 3C and Table S2). No significant difference in other characteristics (*p* > 0.05). In addition, patients in cluster 1 with higher fibrosis content showed a better outcome (*p* = 0.007; HR = 1.850) and lower probability of faster disease progression (*p* = 0.001; HR = 2.131) (Fig. 3D, E).

### Construction and performance of the clinical and radiomics models

**Fig. 4:**
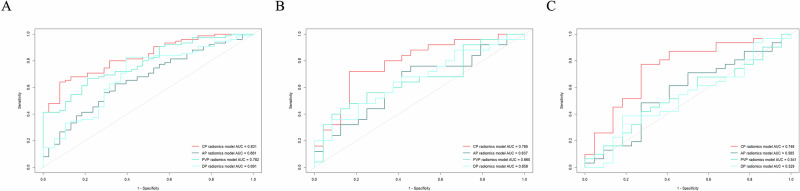
Construction and performance of the radiomics models for predicting different fibrosis groups. Performance of the AP, PVP, DP and CP radiomics model for predicting different fibrosis groups with ROC analysis in the training cohort (**A**), internal test cohort (**B**), and external test cohort (**C**). AUC, area under curve; AP, arterial phase; PVP, portal venous phase; DP, delayed phase; CP, combined phase

In the training cohort, multivariate analyses showed that the PVP (*p* = 0.005; HR = 1.068) and CP Rad-scores (*p* < 0.001; HR = 1.007) were considered the independent predictors for different fibrosis groups (Table 2). After Lasso regression analysis (Fig. S2), 6, 11, 3 and 12 features with nonzero coefficients in AP, PVP, DP and CP were obtained, and used to construct Rad-score, respectively (Appendix E13). The distribution of the radiomics features in each data cohort is shown in Fig. S3. The ROC of the four models for predicting different fibrosis groups in training, internal test and external test cohorts is shown in Fig. 4A–C. The performance of them is displayed in Table 3, compared with the AUC values of the other radiomic models, the CP radiomic model had the best AUC value of the training cohort of 0.831, internal test cohort of 0.785, and external test cohort of 0.746. The calibration curves and the decision curve analysis of the models in the three data cohorts are provided in Fig. S4A–F. Patients in the high CP Rad-score groups of training, internal test and the external test cohorts showed a longer OS (*p* = 0.006; HR = 0.547), (*p* = 0.032; HR = 0.488), (*p* = 0.014; HR = 0.443) and DFS (*p* = 0.016; HR 0.557,), (*p* = 0.026; HR = 0.505), (*p* = 0.006; HR = 0.466) than those in the low Rad-score groups (Fig. S5A–F). Moreover, effect size and confidence interval were calculated to measure the practical significance. The result showed a 0.99 effect size was detected with 97.6% confidence, a maximum of 5% probability of misreporting differences. The result showed that the effect size between the two fibrosis groups was large and the difference in the mean between the two groups was significant, which indicated that the empirical sample size included in this study could support the conclusion.

**Table 2:** Uni- and multivariable logistic regression analysis of variables for their association with different fibrosis groups in patients of training cohort

Characteristic	Univariable analysis		Multivariable analysis	
	OR	*p*-value	OR	*p*-value
Patient demographics				
Gender (male/female)	0.759 (0.318–1.811)	0.534	NA	NA
Age (years)	1.012 (0.971–1.055)	0.563	NA	NA
BMI (kg/m^2^)	1.112 (0.962–1.285)	0.150	NA	NA
Clinical parameters			NA	NA
Hypertension (yes vs no)	0.775 (0.322–1.867)	0.570	NA	NA
Diabetes (yes vs no)	1.398 (0.527–3.713)	0.501	NA	NA
Smoking (yes vs no)	1.422 (0.638–3.166)	0.389	NA	NA
Drinking (yes vs no)	1.081 (0.493–2.367)	0.846	NA	NA
Lymphocyte ratio (%)	0.993 (0.955–1.032)	0.715	NA	NA
Monocyte ratio (%)	1.017 (0.867–1.194)	0.833	NA	NA
Neutrophil ratio (%)	1.007 (0.967–1.048)	0.739	NA	NA
Albumin (g/L)	0.992 (0.975–1.010)	0.389	NA	NA
Globulin (g/L)	1.084 (0.992–1.186)	0.074	NA	NA
Albumin and globulin ratio (%)	0.652 (0.216–1.964)	0.447	NA	NA
CA19-9 (U/mL)	0.999 (0.998–1.000)	0.082	NA	NA
CA12-5 (U/mL)	1.000 (0.991–1.008)	0.940	NA	NA
CEA (ng/mL)	1.033 (0.946–1.127)	0.472	NA	NA
Radiologic features			NA	NA
Location (head vs body and tail)	0.969 (0.439–2.137)	0.937	NA	NA
Tumor necrosis (yes vs no)	0.563 (0.251–1.265)	0.164	NA	NA
Calcification (yes vs no)	2.085 (0.225–19.329)	0.518	NA	NA
Peripancreatic tumor infiltration (yes vs no)	0.311 (0.036–2.680)	0.288	NA	NA
Distal main pancreatic duct dilation (yes vs no)	0.316 (0.086–1.162)	0.083	NA	NA
Adjacent pancreatic parenchymal atrophy (yes vs no)	0.867 (0.392–1.918)	0.724	NA	NA
Arterial involvement (yes vs no)	1.319 (0.580–3.000)	0.509	NA	NA
Venous involvement (yes vs no)	1.253 (0.544–2.886)	0.597	NA	NA
Pathological indicators			NA	NA
Maximal diameter (cm)	1.256 (0.955–1.653)	0.103	NA	NA
Perineural invasion (yes vs no)	1.144 (0.452–2.900)	0.776	NA	NA
Microvascular invasion (yes vs no)	0.788 (0.335–1.851)	0.584	NA	NA
T stage (T1-T2 vs T3-T4)	2.084 (0.886–4.899)	0.092	NA	NA
N stage (N0 vs N1-N2)	2.031 (0.842–4.900)	0.115	NA	NA
Differentiation (well and moderately/poor)	2.035 (0.911–4.547)	0.083	NA	NA
Rad-score				
AP Rad-score	1.049 (1.014–1.084)	0.005	1.000 (0.948–1.054)	0.992
PVP Rad-score	1.060 (1.033–1.088)	< 0.001	1.068 (1.020–1.119)	0.005
DP Rad-score	1.049 (1.018–1.080)	0.002	1.017 (0.972–1.064)	0.470
CP Rad-score	1.006 (1.004–1.009)	< 0.001	1.007 (1.004–1.010)	< 0.001

Data in parentheses are 95% CI. The CP Rad-score was based on the total radiomics model

*CI* confidence interval, *BMI* body mass index, *CA19-9* carbohydrate antigen 19-9, *CA12-5* carbohydrate antigen 12-5, *CEA* carcinoembryonic antigen, *NA* not applicable, *OR* odds ratio, *AP* arterial phase, *PVP* portal venous phase, *DP* delayed phase, *CP* combined phase, *Rad-score* radiomics score

**Table 3:** Performance of the AP, PVP, DP and CP radiomics models for predicting the different fibrosis groups

Model	Feature number	AUC (95% CI)	SEN	SPE	ACC
AP radiomics model	6				
Training cohort		0.661 (0.545–0.764)	0.560	0.684	0.602
Internal test cohort		0.637 (0.478–0.780)	0.560	0.667	0.612
External test cohort		0.565 (0.399–0.722)	0.581	0.591	0.585
PVP radiomics model	11				
Training cohort		0.782 (0.698–0.867)	0.613	0.789	0.673
Internal test cohort		0.660 (0.497–0.801)	0.600	0.583	0.592
External test cohort		0.541 (0.392–0.689)	0.613	0.455	0.547
DP radiomics model	3				
Training cohort		0.691 (0.579–0.788)	0.573	0.711	0.619
Internal test cohort		0.658 (0.505–0.806)	0.520	0.667	0.592
External test cohort		0.529 (0.313–0.625)	0.581	0.409	0.509
CP radiomics model	12				
Training cohort		0.831 (0.754–0.895)	0.707	0.711	0.708
Internal test cohort		0.785 (0.629–0.906)	0.800	0.667	0.735
External test cohort		0.746 (0.618–0.887)	0.871	0.591	0.755

Data in parentheses are 95% CI

*CI* confidence interval, *AUC* area under the receiver operating characteristic curve, *SEN* sensitivity, *SPE* specificity, *ACC* accuracy, *AP* arterial phase, *PVP* portal venous phase, *DP* delayed phase, *CP* combined phase

### Predictive value of the radiomics model for treatment outcomes

**Fig. 5:**
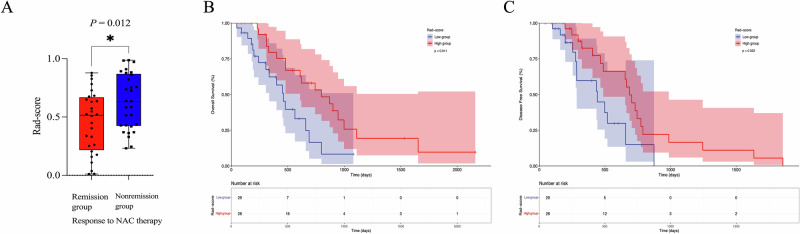
Performance of the CP radiomics model for NAC response and prognosis prediction in the outcome cohort. **A** The relationship between the CP Rad-score and the objective response to NAC. **B** Kaplan–Meier analysis of OS (**B**) and DFS (**C**) according to dichotomized CP Rad-score (low or high, as defined by the 0.5 value of Rad-score)

Baseline characteristics of the patients in the outcome cohort are shown in Table S3. Among the 57 included patients, 28 patients were in the remission group (TRG 0: 4 patients, TRG 1: 4 patients, TRG 2: 20 patients) and 29 patients in the nonremission group (TRG 3: 29 patients). The CP Rad-score was lower in the remission group than that in the nonremission group (0.45 ± 0.27 vs 0.62 ± 0.25; *p* = 0.012) (Fig. 5A). Patients in the high CP Rad-score group showed a longer OS (*p* = 0.011; HR = 0.452) and DFS (*p* = 0.022; HR = 0.468) than those in the low Rad-score group (*p* = 0.027; HR) (Fig. 5B, C). Multivariable analysis revealed that CA19-9 level (*p* = 0.013) and CP Rad-score (*p* = 0.030) were independent predictors for OS (Tables 4, S4), lymphocyte ratio (*p* = 0.021) and CP Rad-score (*p* = 0.022) were independent predictors for DFS (Tables 5, S5).

**Table 4:** Multivariable Cox regression analyses for OS in the outcome cohort

Characteristic	Multivariable analysis	*p*-value
	HR	
Patient demographics		
CA19-9 (U/mL)	1.003 (1.001–1.006)	0.013
CP Rad-score	0.177 (0.037–0.849)	0.030

Data in parentheses are 95% CI

*CI* confidence interval, *OS* overall survival, *CA19-9* carbohydrate antigen 19-9, *HR* hazard ratio, *CP* combined phase

**Table 5:** Multivariable Cox regression analyses for DFS in the outcome cohort

Characteristic	Multivariable analysis	*p*-value
	HR	
Patient demographics		
Lymphocyte ratio (%)	1.044 (1.007–1.083)	0.021
CP Rad-score	0.182 (0.042–0.781)	0.022

Data in parentheses are 95% CI

*CI* confidence interval, *DFS* disease-free survival, *HR* hazard ratio, *CP* combined phase

### Biologic functions associated with radiomics model

**Fig. 6:**
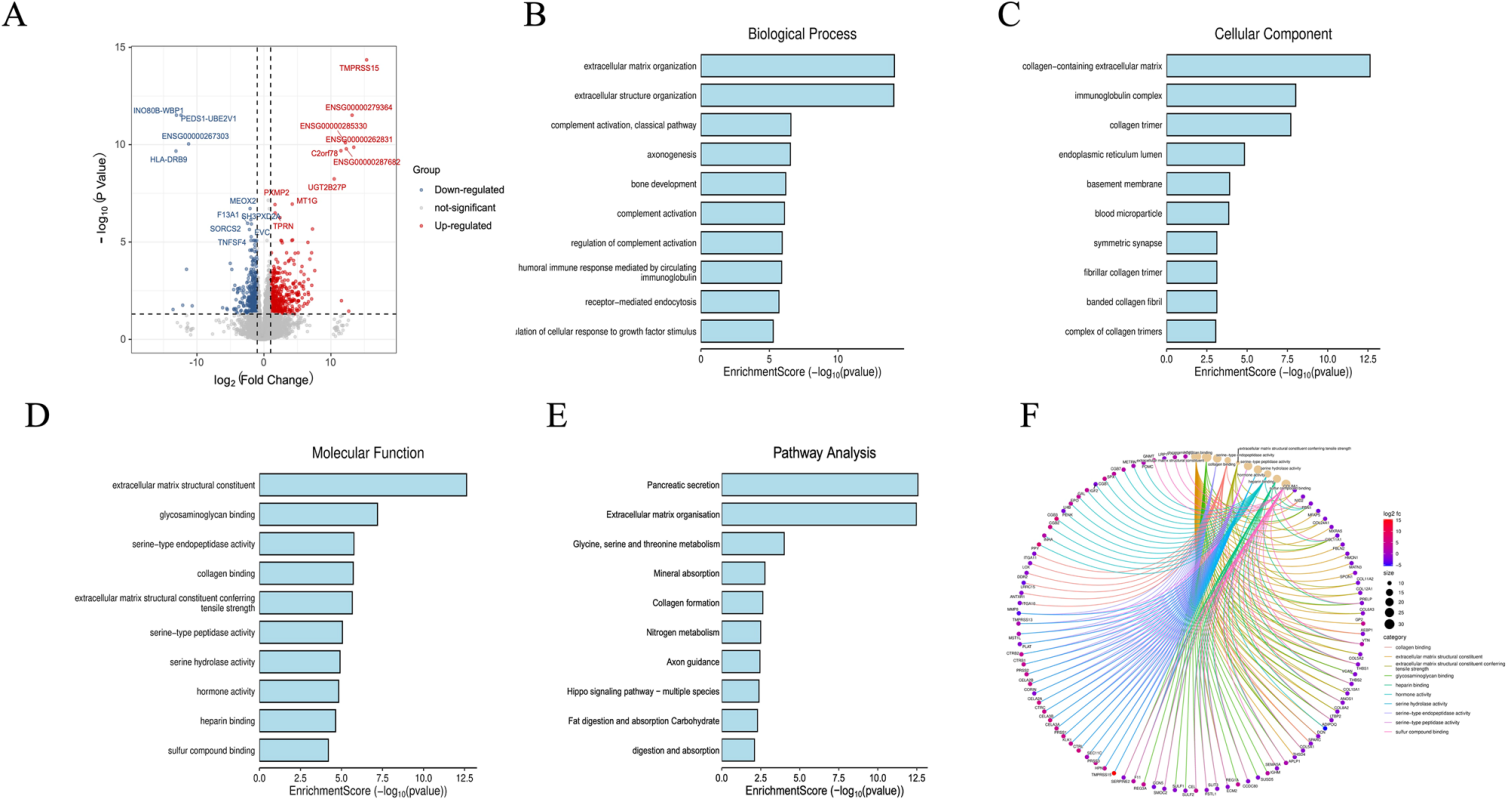
Correlation analysis of the CP Rad-score with molecular data in PDAC. **A** Volcano plot shows the differentially expressed genes in the low Rad-score group compared with the high Rad-score group. Bar plots show the significantly correlated GO biological process (**B**), cellular component (**C**), molecular function (**D**) and KEGG pathway analysis (**E**) associated with the CP Rad-score based on the gene enrichment analysis. **F** Cnet plot indicating the top enriched genes per molecular pathway. GO, Gene Ontology; KEGG, Kyoto Encyclopedia of Genes and Genomes

The patient characteristics in the biological basis cohort are shown in Table S6. Of all 15 patients, 7 are in the low CP Rad-score group and 8 are in the high CP Rad-score group. Forty-six differently expressed genes (DEGs) were identified to be differentially expressed between the two groups and are exhibited in Fig. 6A. The enriched biological processes (Fig. 6B), cellular component (Fig. 6C) and molecular function (Fig. 6D) that significantly correlated with CP Rad-score were linked mainly to the fibrotic production and remodeling processes underlying PDAC. Consistently, the pathways related to fibrosis development, particularly associated with ECM organization, were significantly associated with CP Rad-score (Fig. 6E). Furthermore, among the highly and significantly CP Rad-score correlated genes were multiple ECM genes as well as genes required for ECM assembly and cross-linking (Fig. 6F). The detailed specific gene names are shown in Appendix E14.

## Discussion

Here, we constructed a CP radiomics model to noninvasively predict fibrosis grading in patients with PDAC, with AUC of 0.831, 0.785, and 0.746 in the training set, internal and external test cohorts, respectively. Importantly, our findings based on RNA-seq data uncovered the underlying biological processes (mainly implicated in fibrotic production and remodeling processes) associated with the CP radiomics model.

This study established a clinical-imaging model to preoperatively predict fibrosis grading of PDAC. The results of univariate and multivariate logistic regression analyses showed that only the AP, PVP, DP and CP Rad-scores were independent predictors of different fibrosis grades, and the CP radiomics model showed the best predictive performance. In addition, our study showed no clinical factors associated with different fibrosis grades. Previous studies indicated that there is no consistent conclusion regarding the correlation between different fibrosis grades and clinical characteristics in PDAC [8, 11]. For one thing, we think that the differences between these outcomes may be attributed to differences in diseases, species, and environment. For another, this finding underscores the irreplaceable value of CECT radiomics as a quantitative imaging feature for predicting different PDAC fibrosis grades. Based on this, we further explored the prognosis, NAC efficacy and potential biological significance of the radiomics model.

The benefit of radiomics might arise from the integrative and in-depth information obtained on whole-PDAC, reflecting tissue heterogeneity at different spatial levels. In radiomic terms, spatial tissue heterogeneity is best described by texture features, which identify different image patterns by describing voxel intensities and their spatial arrangement [23]. In our study, most CP radiomics features belonged to the class of texture features or of wavelet transformations thereof. Investigating the added value of CP Rad-score compared with a radiomic score composed only of intensity features further showed that inclusion of such more complex features is crucial for prognostic performance. Our results are in line with previous studies where texture features outperformed first-order (intensity) features for prognostic purposes and where texture features were found to stratify patients according to disease severity [18, 24, 25]. Notably, the features of the CP radiomics model are not simply added up from the selected features in each period, but rather interact and complement each other, thus providing a more comprehensive analysis of tumor characteristics from multiple perspectives.

In recent years, a growing body of evidence has demonstrated that radiomics model holds the potential to address diagnostic ambiguity, monitor response to adjuvant therapies, enhance prognostic models, and even visualize the connection between histologic and biologic features of tumors [26, 27]. The prognostic aspects of our radiomics model were investigated. Fibrosis content has been considered a protective factor of prognosis in many studies and was verified by our study [28, 29]. The possible reason is that fibrosis is tightly arranged around tumor cells in PDAC, which is considered a “fortress-like” barrier that can effectively prevent tumor migration and invasion [30]. Meanwhile, the CP radiomics model constructed in our study also has correlations with OS and DFS in PDAC patients. NAC plays an important role in patients with advanced PDAC, as it can allow surgical resection in a relevant proportion of patients [31]. However, evaluating the efficacy of NAC is a significant challenge in the clinical treatment process. Currently, imaging evaluation criteria based on changes in tumor diameter are commonly used in clinical practice. However, NAC often causes changes at the molecular and cellular levels to achieve downgrading, which does not manifest as a reduction in diameter [32]. Therefore, the evaluation of NAC efficacy in PDAC should shift from morphology to changes in microenvironmental components. Research has found that, in addition to damaged tumor cells, another important indicator of the efficacy evaluation of NAC by pathology is the replacement of tumor cells by interstitial fibrosis. Our study showed that a high Rad-score, likely indicative of the higher fibrosis group, was associated with longer OS and DFS among patients who underwent NAC. These findings are also corroborated by a recent report emphasizing the importance of fibrosis as a pathological assessment of PDAC regression [33].

In contrast to deep learning-based models, which require large datasets and function as “black box” approaches without a clear underlying biological rationale [34], radiomic features were shown to not only correlate with morphological but also with molecular tissue characteristics. This in-depth information provided by radiomics adds a new dimension to previously developed quantitative image analysis [19, 35]. In our biological basis cohort of patients with PDAC, different CP Rad-score groups were closely linked to ECM assembly, fibrotic production and remodeling processes at the molecular level, which demonstrates the potential mechanisms of CP Rad-score-related pathophysiology and the basis for achieving clinical translation. In a word, our work goes further by showing a radiomics link among the CP radomics model, fibrosis grades, NAC response evaluation, prognosis and biological basis, shedding light on risk stratification and personal management for patients with PDAC, with enormous clinical translational potential.

Our study had several limitations. First, a potential selection bias in terms of the inclusion of patients owing to the retrospective design. Second, our radiomics model is built on a surgical cohort; it probably cannot be applied to most PDAC, which are not resected. Next, we will collect patients in layers for research to improve the generalizability of the results. Third, the AUC values of AP, PVP, DP, and CP radiomics models perform excellently on training data, but perform poorly on the validation test set. Additionally, in partial radiomics models, the 95% CI span of AUC is quite large. This possibly due to small sample size, high data noise or uneven distribution of sample size within subgroups, which on the one hand could lead to overfitting of the model, resulting in significant differences in AUC, and on the other hand affects the stability of statistical results, thereby increasing the 95% confidence interval [36, 37]. Moreover, owing to the low incidence rate of PDAC and the low NAC conversion rate of advanced PDAC (about 20%), the sample sizes of the outcome and biological basis cohorts are relatively small. In the future study, we will further expand the sample size to solve the above problems and make the model have more convincing, comprehensive results and higher generalization ability.

## Conclusion

In conclusion, the CP CT radiomics model, built using a combination of CECT radiomics features, can noninvasively predict different fibrosis grades in PDAC. Notably, we uncovered the specific pathophysiological processes underlying the CP radiomics model, mainly involving fibrosis-associated. Furthermore, the CP radiomics model may serve as a noninvasive predictor of OS, DFS and response after NAC, which improves upon conventional risk factors and should be validated in further large prospective studies.

## Supplementary information

ELECTRONIC SUPPLEMENTARY MATERIAL

## Data Availability

The data are available from the corresponding author on reasonable request.

## Abbreviations

CECT: Contrast-enhanced computed tomography
DFS: Disease-free survival
NAC: Neoadjuvant chemotherapy
OS: Overall survival
PDAC: Pancreatic ductal adenocarcinoma
RNA-seq: RNA sequencing
