# Supplementary material for: CT-based radiomics models decode fibrosis content and molecular differences in pancreatic ductal adenocarcinoma: a multi-institutional study
Source: Insights Imaging. 2025 Sep 12;16:190. doi: 10.1186/s13244-025-02036-z (PMC12431998; doi:10.1186/s13244-025-02036-z)
Supplement: Supplementary file 1 — ELECTRONIC SUPPLEMENTARY MATERIAL [file 13244_2025_2036_MOESM1_ESM.pdf]

CT-based radiomics models decode fibrosis content and molecular differences in pancreatic ductal adenocarcinoma: a multi-institutional study

ELECTRONIC SUPPLEMENTARY MATERIAL

Appendix E1: The inclusion and exclusion criteria for surgical resection patients

The inclusion criteria were as follows: (a) surgically resected tumors confirmed as PDAC by postoperative pathology; (b) standard contrast enhanced computed tomography (CECT) performed within 2 weeks before surgery. The exclusion criteria were as follows: (a) incomplete three-phase axial thin-layer reconstruction of CECT images; (b) poor-quality radiologic or pathologic images; (c) patients undergoing other treatments before surgery; (d) surgery-related deaths (died within 90 days after surgery).

Appendix E2: The inclusion and exclusion criteria for outcome cohort patients

The inclusion criteria were as follows: (a) patients who received NAC treatment before surgery; (b) preoperative puncture pathology confirmed PDAC; (c) standard CECT performed within 2 weeks before surgery. The exclusion criteria were as follows: (a) incomplete three-phase axial thin-layer reconstruction of CECT images; (b) poor-quality radiologic or pathologic images; (c) patients undergoing treatments other than NAC before surgery; (d) surgery-related deaths (died within 90 days after surgery).

Appendix E3: Detailed NAC regimens for outcome cohort patients

All patients in the outcome cohort received NAC according to the guidelines of the National Comprehensive Cancer Network[1]. Among them, 47 patients received 2 to 8 cycles of the AG regimen (gemcitabine 1000-1250 mg/m^2 ivgtt, day 1, 8 and albumin-bound paclitaxel 125 mg/m^2 ivgtt, day 1, 8; repeated every 3 weeks); 7 patients received 1 to 7 cycles of the GS regimen (gemcitabine 1000 mg/m^2 , ivgtt, day 1, 8 and S-1 80-120 mg/d, orally (bid), day 1-14, repeated every 3 weeks) , and 3 patients received 2 to 6 cycles of the FOLFIRINOX regimen (oxaliplatin 85 mg/m^2 ivgtt, day 1; irinotecan180 mg/m^2 ivgtt, day 1; calcium folinate 400 mg/m^2 ivgtt, day 1; 5-FU 400 mg/m^2 ivgtt, day 1; followed by 5-FU 2400 mg/m^2 continuous infusion 46 h, repeated every 2 weeks).

Appendix E4: The inclusion and exclusion criteria for biological basis cohort patients

The inclusion criteria were as follows: (a) surgically resected tumors confirmed as PDAC by postoperative pathology; (b) standard CECT performed within 2 weeks before surgery. The exclusion criteria were as follows: (a) intraoperative detection of liver micrometastases not visible on preoperative CT images; (b)incomplete three-stage axial thin-layer reconstruction of CECT images; (c) poor-quality radiologic; (d) patients undergoing other treatments before surgery; (e) severely degraded tumor specimens for which RNA-seq could not be performed.

Appendix E5: CT scanning protocol

All patients fasted for 6-8 h and were then given 800-1000 ml of warm water 20 min before the examination to ensure better image contrast. The CT examinations were performed while holding the breath, with the patient in the supine position. Scans were performed on Philips Healthcare (Brilliance iCT, Netherlands), Siemens Healthineers (Somatom Force CT, Germany) or GE Healthcare CT (Revolution CT, America) scanners with 16-320 channels. Plain CT images were acquired first, after that, patients received approximately 1.3-1.5 mL/kg body weight bolus of contrast material iodixanol (Ultravist 300, Bayer, Germany) through the cubital vein at a rate of 2.5-3.0 mL/s. CT scans of the arterial phase (AP), portal venous phase (PVP) and delayed phase (DP) were performed at 20-25 s, 60-75 s, and 150-180 s, after injection of the contrast agent, respectively. Scanning parameters were as follows: tube voltage of 100 or 120 kVp; tube current of 150-200 mAs or automatic tube current modulation technology; the field of view, 350 × 350-500 × 500 mm; matrix, 512 × 512; slice thickness, 5 mm and CT scans were reconstructed to 1 mm slice thickness.

Appendix E6: Features of image analysis

Radiological features including tumor location; tumor necrosis; tumor calcification; distal main pancreatic duct dilation (≥ 3 mm)[2]; peripancreatic tumor infiltration (tumor infiltration into the area around the pancreas); adjacent pancreatic parenchymal atrophy (≤ 7 mm at the pancreatic body)[3]; arterial (common hepatic artery, celiac axis, superior mesenteric artery) or vein vascular (portal vein or superior mesenteric vein) involvement. Evaluation of the main arterial involvement was based on the presence of abutment or encasement. Venous involvement was characterized by either no tumor contact or contact of ≤ 180 degrees without vein contour irregularity[4].

Appendix E7: The data for clinicopathological variables and detailed patients grouping after NAC

Clinical and conventional pathological data including gender, age, body mass index (BMI), hypertension, diabetes, smoking, drinking, lymphocyte ratio, monocyte ratio, neutrophil ratio, albumin, globulin, albumin and globulin ratio, antigen 19-9 (CA 19-9), antigen 12-5 (CA 12-5) carcinoembryonic antigen (CEA) levels, histologic grade, maximal tumor diameter, T stage, N stage, microvascular invasion and perineural invasion. TRG system was graded as follows:

score 0, no viable cancer cells; score 1, single cells or rare small groups of cancer cells; score 2, residual cancer with evident tumour regression, but more than single cells or rare small groups of cancer cells and score 3, extensive residual cancer with no evident tumor regression. Final, patients who underwent surgical resection after NAC were divided into two groups: the remission group (score 0, score 1 and score 2) and the nonremission group (score 3).

Appendix E8: The measurement of fibrosis content

All specimens were fixed in 10% neutral formaldehyde solution, dehydrated conventionally, embedded in paraffin,

and cut into sections of 4-μm thick. Five high-magnification fields (×400) with the highest tumor content were selected according to hematoxylin and eosin (H&E) staining, and Masson staining was used to measure the fibrosis content. Image-Pro Plus software (Media Cybernetics, Inc) was used to quantitatively analyze fibrosis and full-field pixel area. The proportion of fibrotic pixels relative to the total number of pixels was defined as fibrosis content.

Appendix E9: Image segmentation and radiomic feature extraction

Regions of interest (ROI) were manually delineated section by section for the index tumors on the AP, PVP, and DP images and were fused to generate the volumes of interest, as shown in Figure S1. During the process of delineating ROI, minimize the avoidance of necrotic, cystic, hemorrhage, calcification of the lesion, as well as normal pancreatic tissue, blood vessels, bile ducts, and adipose tissue around the lesion.

Before radiomics feature extraction, the voxel dimensions were isotopically resampled to 1 × 1 × 1 mm (x-, y-, and z-axes) by a linear interpolation algorithm to correct for acquisition-related voxel resolution variations. Radiomic features were extracted using the Python package radiomics-based open-source software (version 2.2.3, https://mics.radcloud.cn/ - /login). The values of the radiomic features were normalized using the z-score method.

Appendix E10: RNA-seq analysis

There were 33 patients with PDAC included in the biological basis cohort. Of which, 6 patients were excluded due to intraoperative detection of liver micrometastases that were not visible on CT imagines, 3 patients were excluded owing to receive other treatments before surgery, 4 patients were excluded due to incomplete preoperative three-phase axial thin-layer reconstruction of CECT images, 3 patients were excluded owing to poor-quality radiologic images and 2 were excluded due to severe PDAC sample degradation. Finally, 15 patients with both available AP, PVP, DP CT images and RNA-seq data were included.

15 Patients in the biological basis cohort were stratified into low-or high-score groups based on the model score threshold of 0.5. RNA was extracted from the PDAC tissue specimens. All cells from the tumor specimens were used to perform fibrosis related identification based on RNA-seq level. Gene expression levels were calculated using the fragments per kilobase of transcript per million (FPKM) format, whereas Differentially Expressed Genes (DEGs) were identified using the count format. We used the “DESeq” package for the analysis of fibrosis-related DEGs, with thresholds set at an adjusted *P* value < 0.05, |log2 (fold change) | > 1 and a false-discovery rate (FDR) < 0.05 were set. For fibrosis related DEGs, Gene Ontology (GO) and Kyoto Encyclopedia of Genes and Genomes (KEGG) functional enrichment analyses were performed using the “clusterProfiler” package with thresholds of P value < 0.05 and and q value < 0.05. Simultaneously, fibrosis-related genes were enriched using ClueGO in Cytoscape software (version 3.8.0) with default parameters[3,5].

Appendix E11: Estimation of sample size

For the estimation of the sample size for radiomics model construction, at least 38 patients (15 patients with low fibrosis content and 23 with high fibrosis content) were required in each dataset according to the following input and assumption: power, 0.9; two-sided significance level, 0.05; alternative hypothesis of the AUC, 0.800 compared with the null hypothesis of the AUC, 0.500, and an allocation ratio of sample sizes in the high and low groups of 1.5. In our study, the training cohort, internal test cohort, and external test cohort included 113 (38 with low fibrosis and 75 with high fibrosis), 49 (24 with low fibrosis and 25 with high fibrosis), and 53 patients (22 with low fibrosis and 31 with high fibrosis), respectively. Therefore, it was sufficient to detect an AUC different from 0.500 with 80% power if the true AUC was > 0.800[6].

Appendix E12: Packages of R Software

The extraction of radiomic features was implemented using R Software (version 3.6.1). K-means clustering, Lasso regression for feature selection, logistic regression for modeling as well as calibration curves, ROC curve analysis and AUC calculation were all performed using scikit-learn 1.1.3. Student t test, Mann-Whitney U test, Kruskal-Wallis H test, Chi-square test or Fisher's exact test were conducted using SciPy 1.10.0.

Appendix E13: Formula of the Rad-score

AP Rad-score = 0.273*wavelet-LHL_glcm_Correlation_A + 0.296*wavelet-LHH_glcm_Correlation_A + 0.283*exponential_glrlm_RunLengthNonUniformity_A -0.207*wavelet-LHL_glcm_Imc2_A - 0.339*wavelet-HHL_glcm_Contrast_A -0.433*wavelet-HHL_glrlm_GrayLevelNonUniformityNormalized_A

PVP Rad-score = 0.461*wavelet-LLH_glcm_JointAverage_V -0.629*wavelet-LLL_ngtdm_Contrast_V + 0.292*wavelet-HHL_glcm_Correlation_V -0.225*wavelet-HHL_glcm_MCC_V -0.877*wavelet-LHL_glcm_Imc1_V -0.9601*wavelet-LHL_firstorder_Variance_V + 0.487*wavelet-HHH_glcm_Imc1_V - 0.6012*wavelet-LHL_glrlm_RunVariance_V + 0.839*wavelet-HLH_firstorder_Maximum_V + 0.2178*original_shape_Maximum2DDiameterSlice_V + 0.4012*wavelet-HHL_glcm_ClusterProminence_V

DP Rad-score = 0.585*wavelet-HLH_glszm_ZoneEntropy_D -0.179*wavelet-HHL_glcm_MCC_D + 0.406*wavelet-HHL_glcm_Correlation_D

CP Rad-score = 0.447*wavelet-HHL_glcm_Correlation_A+0.494*wavelet-LLH_glcm_JointAverage_V-0.882*wavelet-LLL_ngtdm_Contrast_V-0.515*wavelet-HHL_glcm_MCC_V+ 1.045*wavelet-HLH_glszm_ZoneEntropy_D -0.157*wavelet-LHL_glcm_Imc1_V-0.879*wavelet-HHL_glcm_Imc1_D+ 0.491*wavelet-LHH_glcm_Correlation_A-0.0279*wavelet-HHL_ngtdm_Contrast_A-0.333*wavelet LHL_firstorder_Variance_V-0.2892*wavelet-LHL_glrlm_RunVariance_V +0.001*exponential_glrlm_RunLengthNonUniformity_A

Appendix E14: Specific gene names

Among the highly and significantly Rad-score correlated genes were multiple ECM genes, such as collagen 8A1 (COL8A1; FDR < 0.001), collagen 10A1 (COL10A1; FDR = 0.018), collagen 11A1 (COL11A1; FDR =0.004), collagen 12A1 (COL12A1; FDR =0.008), collagen 5A1 (COL5A1; FDR = 0.043), collagen 24A1 (COL24A1; FDR =0.003), ECM2 (FDR =0.011) and FERM Domain Containing

Kindlin 2 (FERMT2; FDR=0.038), as well as genes required for ECM assembly and cross-linking, including members of the lysyl oxidase family, such as LOXL1 (FDR =0.013). In addition, the genes involved in transforming growth factor (TGF)-β activation, including latent-TGF-β-binding protein 2 (LTBP2; FDR =0.026), was of great significance for the CP Rad-score.

Appendix Reference:

1. Tempero MA, Malafa MP, Al-Hawary M et al (2021) Pancreatic Adenocarcinoma, Version 2.2021, NCCN Clinical Practice Guidelines in Oncology. J Natl Compr Canc Netw 19:439-457. https://doi.org/10.6004/jnccn.2021.0017
2. Shi Y, Liu Y, Gao F et al (2018) Pancreatic Stiffness Quantified with MR Elastography: Relationship to Postoperative Pancreatic Fistula after Pancreaticoenteric Anastomosis. Radiology 288:476-484. https://doi.org/10.1148/radiol.2018170450
3. Tirkes T, Shah ZK, Takahashi N et al (2019) Reporting Standards for Chronic Pancreatitis by Using CT, MRI, and MR Cholangiopancreatography: The Consortium for the Study of Chronic Pancreatitis, Diabetes, and Pancreatic Cancer. Radiology 290:207-215. https://doi.org/10.1148/radiol.2018181353
4. Al-Hawary MM, Francis IR, Chari ST et al (2014) Pancreatic ductal adenocarcinoma radiology reporting template: consensus statement of the society of abdominal radiology and the american pancreatic association. Gastroenterology 146:291-304.e291. https://doi.org/10.1053/j.gastro.2013.11.004
5. Xia TY, Zhou ZH, Meng XP et al (2023) Predicting Microvascular Invasion in Hepatocellular Carcinoma Using CT-based Radiomics Model. Radiology 307:e222729. https://doi.org/10.1148/radiol.222729
6. Feng Z, Li H, Liu Q et al (2023) CT Radiomics to Predict Macrotrabecular-Massive Subtype and Immune Status in Hepatocellular Carcinoma. Radiology 307:e221291. https://doi.org/10.1148/radiol.221291

Table S1 The clinical, radiological and pathological characteristics of internal and external test cohorts

Characteristics		Internal test cohort (n = 49)			External test cohort (n = 53)	
Patient demographics	Low fibrosis group (n=24)	High fibrosis group (n=25)	*P* value	Low fibrosis group (n=22)	High fibrosis group (n=31)	*P* value
Gender			0.686			0.272
Male	16 (66.67)	18 (72.00)		14 (63.64)	15 (48.39)	
Female	8 (33.33)	7 (28.00)		8 (36.36)	16 (51.61)	
Age (years) *	61.08 ± 10.93	58.00 ±12.59	0.365	58.70 ± 9.97	64.67 ± 8.84	0.036
BMI (kg/m^2) *	24.44 ± 3.52	22.15 ± 2.95	0.044	23.66 ± 3.99	23.68 ± 3.29	0.983
Clinical parameters						
Hypertension			0.628			0.872
Yes	8 (33.33)	10 (40.00)		9 (40.91)	12 (38.71)	
No	16 (66.67)	15 (60.00)		13 (59.09)	19 (61.29)	
Diabetes			0.456			0.246
Yes	7 (29.17)	5 (20.00)		9 (40.91)	8 (25.81)	
No	17 (70.83)	20 (80.00)		13 (59.09)	23 (74.19)	
Smoking			0.666			0.697
Yes	11 (45.83)	13 (52.00)		6 (27.27)	10 (32.26)	
No	13 (54.17)	12 (48.00)		16 (72.73)	21 (67.74)	
Drinking			0.879			0.632
Yes	11 (45.83)	12 (48.00)		7 (31.82)	8 (25.81)	
No	13 (54.17)	13 (52.00)		15 (68.18)	23 (74.19)	
Lymphocyte ratio (%) *	24.90 ± 7.93	24.58 ± 8.22	0.893	29.82 ± 8.23	27.65 ± 9.73	0.461
Monocyte ratio (%) *	8.24 ± 3.07	6.84 ± 2.66	0.093	8.32 ± 2.13	7.04 ± 1.96	0.056
Neutrophil ratio (%) *	63.58 ± 9.43	66.19 ± 9.86	0.348	58.15 ± 8.15	62.23 ± 9.96	0.176
Albumin (g/L) *	41.67 ± 7.67	41.57 ± 4.59	0.957	40.79 ± 6.19	39.96± 6.64	0.683
Globulin (g/L) *	24.71 ± 4.43	27.75 ± 4.58	0.024	25.84 ± 4.79	23.63 ± 4.99	0.157
Albumin and Globulin ratio (%) *	1.69 ± 0.34	1.54 ± 0.30	0.118	1.59 ± 0.30	1.72 ± 0.26	0.141
CA19-9 (U/mL) *	369.12 ± 345.68	268.61 ± 336.16	0.323	491.35 ± 356.27	360.56 ± 345.31	0.212
CA12-5 (U/mL) *	21.03 ± 21.86	21.91 ± 21.04	0.895	17.22 ± 13.95	22.86 ± 17.63	0.267
CEA (ng/mL) *	5.52 ± 8.11	5.64 ± 7.39	0.961	4.95 ± 3.11	6.12 ± 9.42	0.629
Radiologic features						
Location			0.187			0.688
Head	16 (66.67)	12 (48.00)		13 (59.09)	20 (64.52)	
Body & Tail	8 (33.33)	13 (52.00)		9 (40.91)	11 (35.48)	
Tumor necrosis			0.058			0.833
Yes	17 (70.83)	11 (44.00)		10 (45.45)	15 (48.39)	
No	7 (29.17)	14 (56.00)		12 (54.56)	16 (51.61)	
Calcification			0.322			0.767
Yes	0 (0)	1 (4.00)		1 (4.55)	2 (6.45)	
No	24 (100)	24 (96.00)		21 (95.45)	29 (93.54)	
Peripancreatic tumor			0.171			0.184

infiltration						
Yes	23 (95.83)	21 (84.00)		18 (81.82)	29 (93.54)	
No	1 (4.17)	4 (16.00)		4 (18.18)	2 (6.45)	
Distal main pancreatic duct dilation			0.524			0.845
Yes	20 (83.33)	19 (76.00)		18 (81.82)	26 (83.87)	
No	4 (16.67)	6 (24.00)		4 (18.18)	5 (16.13)	
Adjacent pancreatic parenchymal atrophy			0644			0.168
Yes	9 (37.50)	11 (44.00)		12 (54.56)	11 (35.48)	
No	15 (62.50)	14 (56.00)		10 (45.45)	20 (64.52)	
Arterial invasion			0.845			0.573
Yes	16 (66.67)	16 (64.00)		14 (63.64)	22 (70.97)	
No	8 (33.33)	9 (36.00)		8 (36.36)	9 (29.03)	
Venous invasion			0.684			0.623
Yes	14 (58.33)	16 (64.00)		12 (54.56)	19 (61.29)	
No	10 (41.67)	9 (36.00)		10 (45.45)	12 (38.71)	
Pathological indicators						
Maximal diameter (cm) *	4.25 ± 2.07	3.70 ± 1.28	0.270	3.29 ± 1.36	3.42 ± 1.60	0.759
Perineural invasion			0.483			0.252
Yes	14 (58.33)	17 (68.00)		18 (81.82)	21 (67.74)	
No	10 (41.67)	8 (32.00)		4 (18.18)	10 (32.26)	
Microvascular invasion			0.470			0.518
Yes	8 (33.33)	6 (24.00)		9 (40.91)	10 (32.26)	
No	16 (66.67)	19 (76.00)		13 (59.09)	21 (67.74)	
T stage			0.858			0.075
T1-2	15 (62.50)	15 (60.00)		19 (86.36)	20 (64.52)	
T3-4	9 (37.50)	10 (40.00)		3 (13.64)	11 (35.48)	
N stage						0.409
N0	16 (66.67)	18 (72.00)	0.686	14 (63.64)	23(74.19)	
N1-2	8 (33.33)	7 (28.00)		8 (36.36)	8(25.81)	
Differentiation			0.686			0.003
Well & moderately	16 (66.67)	18 (72.00)		8 (36.36)	24 (77.42)	
Poor	8 (33.33)	7 (28.00)		14 (63.64)	7 (22.58)	

Note. —Unless indicated otherwise, data are number of patients, and data in parentheses are percentages.

BMI, body mass index; CA19-9, carbohydrate antigen 19-9; CA12-5, carbohydrate antigen 12-5; CEA, carcinoembryonic antigen.

* Data are means ± SDs.

Table S2: Associations of the identified patients' clusters based on their radiomics features with clinical, radiological and pathological characteristics for the training cohort

Characteristic	Cluster 1 (n=46)	Cluster 2 (n=67)	*P* value
Patient demographics			
Gender			0.442
Male	34 (73.91)	45 (67.16)	
Female	12 (26.09)	22 (32.84)	
Age (years) *	61.93 ± 9.11	59.97 ± 9.58	0.277
BMI (kg/m^2) *	22.24 ± 2.42	23.74 ± 3.13	0.010
Clinical parameters			
Hypertension			0.932
Yes	12 (26.09)	17 (25.37)	
No	34 (73.91)	50 (74.63)	
Diabetes			0.587
Yes	9 (19.57)	16 (23.88)	
No	37 (80.43)	51 (76.12)	
Smoking			0.034
Yes	25 (54.35)	23 (34.33)	
No	21 (45.65)	44 (65.67)	
Drinking			0.929
Yes	23 (50.00)	29 (43.28)	
No	23 (50.00)	38 (56.72)	
Lymphocyte ratio (%) *	29.30 ± 11.16	26.42 ± 9.20	0.141
Monocyte ratio (%) *	7.34± 1.79	7.63 ± 2.88	0.546
Neutrophil ratio (%) *	61.74 ± 10.10	63.12 ± 9.79	0.475
Albumin (g/L) *	40.84 ± 6.04	42.47± 4.58	0.111
Globulin (g/L) *	25.64± 5.19	25.91 ± 4.35	0.772
Albumin and Globulin ratio (%) *	1.65 ± 0.39	1.67 ± 0.34	0.713
CA19-9 (U/mL) *	390.50 ± 347.86	399.82 ± 377.26	0.896
CA12-5 (U/mL) *	37.56 ± 61.05	31.59 ± 43.57	0.581
CEA (ng/mL) *	4.72 ± 4.61	5.20 ± 6.28	0.668
Radiologic features			
Location			0.265
Head	24 (52.17)	42 (62.69)	
Body & tail	22 (47.83)	25 (37.31)	
Tumor necrosis			0.255
Yes	29 (63.04)	35 (52.24)	
No	17 (36.96)	32 (47.76)	
Calcification			0.335
Yes	1 (2.17)	4 (5.97)	
No	45 (97.83)	63 (94.03)	
Peripancreatic tumor infiltration			0.142
Yes	45 (97.83)	61 (91.04)	

No	1 (2.17)	6 (8.96)	
Distal main pancreatic duct dilation			0.246
Yes	36 (78.26)	58 (86.57)	
No	10 (21.74)	9 (13.43)	
Adjacent pancreatic parenchymal atrophy			0.790
Yes	19 (41.30)	26 (38.81)	
No	27 (58.70)	41 (61.19)	
Arterial involvement			0.039
Yes	36 (78.26)	40 (59.70)	
No	10 (21.74)	27 (40.30)	
Venous involvement			0.468
Yes	30 (78.26)	48 (71.64)	
No	16 (21.74)	19 (28.36)	
Pathological indicators			
Maximal diameter (cm) *	3.55 ± 1.38	4.54 ± 2.00	0.002
Perineural invasion			0.997
Yes	35 (76.09)	51 (76.12)	
No	11 (23.91)	16 (23.88)	
Microvascular invasion			0.991
Yes	13 (28.26)	19 (28.36)	
No	33 (71.74)	48 (71.64)	
T stage			0.122
T1-2	25 (54.35)	46 (68.66)	
T3-4	21 (45.65)	21 (31.34)	
N stage			0.552
N0	32 (69.57)	43 (64.18)	
N1-2	14 (30.43)	24 (35.82)	
Differentiation			0.501
Well & Moderately	31 (67.39)	41 (61.19)	
Poor	15 (32,61)	26 (38.81)	
Fibrosis content (%) *	64.12 ± 16.92	50.98 ± 19.22	<0.001

Note. —Unless indicated otherwise, data are number of patients, and data in parentheses are percentages.

BMI, body mass index; CA19-9, carbohydrate antigen 19-9; CA12-5, carbohydrate antigen 12-5; CEA, carcinoembryonic antigen.

* Data are means ± SDs.

Table S3 Baseline characteristics of patients in outcomes test cohort

Characteristics	Low Score Group (n = 23)	High Score Group (n = 34)	*P* value
Patient demographics			
Gender (Male)	13 (56.52)	20 (58.82)	0.863
Age (years) *	58.17 ± 10.06	56.15 ± 8.42	0.414
BMI (kg/m^2) *	23.37 ± 3.58	23.42 ± 2.69	0.956
Clinical parameters			
Hypertension (Yes)	6 (26.09)	11 (32.35)	0.612
Diabetes (Yes)	4 (17.39)	9 (26.47)	0.423
Smoking (Yes)	10 (43.48)	9 (26.47)	0.181
Drinking (Yes)	9 (39.13)	11 (32.35)	0.599
Lymphocyte ratio (%) *	24.13 ± 8.81	27.98 ± 11.27	0.223
Monocyte ratio (%) *	8.40 ± 3.51	7.62 ± 2.26	0.370
Neutrophil ratio (%) *	56.87 ± 22.11	58.38 ± 20.72	0.813
Albumin (g/L) *	43.86 ± 7.65	42.67 ± 3.69	0.497
Globulin (g/L) *	28.83 ± 6.51	25.02 ± 4.43	0.046
Albumin and Globulin ratio (%) *	3.61 ± 6.78	1.77 ± 0.42	0.188
CA19-9 (U/mL) *	168.87 ± 174.39	62.43 ± 81.25	0.010
CA12-5 (U/mL) *	43.84 ± 94.40	18.57 ± 13.72	0.220
CEA (ng/mL) *	9.31 ± 15.47	4.15 ± 4.09	0.133
NAC scheme (AG)	21 (91.30)	26 (76.47)	0.149
Radiologic features			
Location (Head)	10 (43.48)	20 (58.82)	0.255
Tumor necrosis (Yes)	14 (60.87)	12 (35.29)	0.057
Calcification (Yes)	2 (8.70)	1 (2.94)	0.340
Peripancreatic tumor infiltration (Yes)	18 (78.26)	22 (64.71)	0.272
Distal main pancreatic duct dilation (Yes)	20 (86.96)	25 (73.53)	0.223
Adjacent pancreatic parenchymal atrophy (Yes)	13 (56.52)	20 (58.82)	0.863
Arterial invasion (Yes)	7 (30.43)	10 (29.41)	0.934
Venous invasion (Yes)	9 (39.13)	15 (44.12)	0.708
Pathological indicators			
Maximal diameter (cm) *	3.89 ± 1.87	2.88 ± 1.12	0.025
Perineural invasion (Yes)	19 (82.61)	21 (61.76)	0.091
Microvascular invasion (Yes)	6 (26.09)	11 (32.35)	0.612
T stage (T1-2)	19 (82.61)	26 (76.47)	0.577
N stage (N0)	18 (78.26)	29 (85.29)	0.493
Differentiation (Poor)	7 (30.43)	13 (38.24)	0.545
Fibrosis content (%)	46.82 ± 17.61	61.07 ± 17.07	0.002

Note. —Unless indicated otherwise, data are number of patients, and data in parentheses are percentages.

BMI, body mass index; CA19-9, carbohydrate antigen 19-9; CA12-5, carbohydrate antigen 12-5; CEA, carcinoembryonic antigen; AG, albumin-bound paclitaxel and gemcitabine.

* Data are means ± SDs.

Table S4: Uni- and multivariable cox regression analyses for OS in the outcome cohort

Characteristic	Univariable Analysis		Multivariable Analysis	
	HR	*P* value	HR	*P* value
Patient demographics				
Gender (Male/Female)	0.692 (0.324-1.475)	0.340	NA	NA
Age (Years)	0.966 (0.923-1.011)	0.140	NA	NA
BMI (kg/m^2)	0.981 (0.865-1.112)	0.764	NA	NA
Clinical parameters			NA	NA
Hypertension (Yes vs No)	0.470 (0.178-1.244)	0.128	NA	NA
Diabetes (Yes vs No)	0.963 (0.386-2.400)	0.936	NA	NA
Smoking (Yes vs No)	0.731 (0.319-1.675)	0.458	NA	NA
Drinking (Yes vs No)	1.188 (0.541-2.608)	0.668	NA	NA
Lymphocyte ratio (%)	1.022 (0.990-1.055)	0.186	NA	NA
Monocyte ratio (%)	1.087 (1.012-1.168)	0.022	1.043 (0.963-1.129)	0.301
Neutrophil ratio (%)	1.009 (0.987-1.031)	0.442	NA	NA
Albumin (g/L)	1.048 (0.974-1.106)	0.083	NA	NA
Globulin (g/L)	1.004 (0.919-1.096)	0.935	NA	NA
Albumin and Globulin ratio (%)	1.033 (0.950-1.123)	0.447	NA	NA
CA19-9 (U/mL)	1.003 (1.000-1.005)	0.025	1.003 (1.001-1.006)	0.013
CA12-5 (U/mL)	1.205 (0.781-1.859)	0.399	NA	NA
CEA (ng/mL)	1.010 (0.995-1.027)	0.199	NA	NA
Radiologic features			NA	NA
Location (Head vs Body & Tail)	0.568 (0.263-1.227)	0.150	NA	NA
Tumor necrosis (Yes vs No)	0.592 (0.140-2.506)	0.476	NA	NA
Calcification (Yes vs No)	3.026 (0.893-10.252)	0.075	NA	NA
Peripancreatic tumor infiltration (Yes vs No)	0.737 (0.308-1.765)	0.493	NA	NA
Distal main pancreatic duct dilation (Yes vs No)	1.298 (0.593-2.844)	0.514	NA	NA
Adjacent pancreatic parenchymal atrophy (Yes vs No)	0.975 (0.445-2.134)	0.949	NA	NA
Arterial involvement (Yes vs No)	0.606 (0.254-1.447)	0.259	NA	NA
Venous involvement (Yes vs No)	0.603 (0.273-1.333)	0.212	NA	NA
Pathological indicators			NA	NA
Maximal diameter (cm)	1.051 (0.821-1.346)	0.693	NA	NA
Perineural invasion (Yes vs No)	1.393 (0.572-3.393)	0.465	NA	NA
Microvascular invasion (Yes vs No)	0.603 (0.178-2.040)	0.416	NA	NA
T stage (T1-T2 vs T3-T4)	0.753 (0.281-2.019)	0.573	NA	NA
N stage (N0 vs N1-N2)	1.169 (0.402-3.397)	0.775	NA	NA
Differentiation (Well & moderately/Poor)	0.939 (0.429-2.056)	0.875	NA	NA
CP Rad-score	0.184 (0.046-0.733)	0.016	0.177 (0.037-0.849)	0.030

Note. -Data in parentheses are 95% CI. CI, confidence interval; OS, overall survival; BMI, body mass index; CA19-9, carbohydrate antigen 19-9; CA12-5, carbohydrate antigen 12-5; CEA, NA = not applicable, HR = hazard ratio; CP, combined phase.

Table S5 Uni- and multivariable cox regression analyses for DFS in the outcome cohort

Characteristic	Univariable Analysis		Multivariable Analysis	
	HR	*P* value	HR	*P* value
Patient demographics				
Gender (Male/Female)	1.244 (0.586-2.640)	0.570	NA	NA
Age (Years)	1.018 (0.958-1.082)	0.565	NA	NA
BMI (kg/m^2)	0.904 (0.805-1.013)	0.083	NA	NA
Clinical parameters			NA	NA
Hypertension (Yes vs No)	0.696 (0.324-1.498)	0.354	NA	NA
Diabetes (Yes vs No)	1.034 (0.450-2.378)	0.937	NA	NA
Smoking (Yes vs No)	2.141 (0.945-4.852)	0.068	NA	NA
Drinking (Yes vs No)	1.592 (0.713-3.556)	0.257	NA	NA
Lymphocyte ratio (%)	1.043 (1.009-1.079)	0.013	1.044 (1.007-1.083)	0.021
Monocyte ratio (%)	1.079 (0.935-1.244)	0.298	NA	NA
Neutrophil ratio (%)	1.001 (0.985-1.019)	0.867	NA	NA
Albumin (g/L)	1.040 (0.975-1.109)	0.239	NA	NA
Globulin (g/L)	0.985 (0.892-1.087)	0.765	NA	NA
Albumin and Globulin ratio (%)	1.675 (0.739-3.796)	0.217	NA	NA
CA19-9 (U/mL)	0.998 (0.995-1.001)	0.168	NA	NA
CA12-5 (U/mL)	0.997 (0.905-1.099)	0.952	NA	NA
CEA (ng/mL)	0.769 (0.534-1.107)	0.158	NA	NA
Radiologic features			NA	NA
Location (Head vs Body & Tail)	0.586 (0.283-1.211)	0.149	NA	NA
Tumor necrosis (Yes vs No)	1.678 (0.560-5.034)	0.355	NA	NA
Calcification (Yes vs No)	2.969 (0.375-23.526)	0.303	NA	NA
Peripancreatic tumor infiltration (Yes vs No)	1.715 (0.654-4.500)	0.273	NA	NA
Distal main pancreatic duct dilation (Yes vs No)	1.634 (0.788-3.389)	0.187	NA	NA
Adjacent pancreatic parenchymal atrophy (Yes vs No)	0.975 (0.469-2.024)	0.945	NA	NA
Arterial involvement (Yes vs No)	1.314 (0.636-2.712)	0.461	NA	NA
Venous involvement (Yes vs No)	1.037 (0.508-2.114)	0.921	NA	NA
Pathological indicators			NA	NA
Maximal diameter (cm)	0.962 (0.747-1.237)	0.761	NA	NA
Perineural invasion (Yes vs No)	0.595 (0.279-1.271)	0.180	NA	NA
Microvascular invasion (Yes vs No)	1.150 (0.413-3.199)	0.789	NA	NA
T stage (T1-T2 vs T3-T4)	0.713 (0.299-1.700)	0.445	NA	NA
N stage (N0 vs N1-N2)	1.089 (0.375-3.162)	0.875	NA	NA
Differentiation (Well & moderately/Poor)	1.190 (0.515-2.749)	0.683	NA	NA
CP Rad-score	0.147 (0.035-0.625)	0.009	0.182 (0.042-0.781)	0.022

Note. -Data in parentheses are 95% CI. CI, confidence interval; DFS, disease-free survival; BMI, body mass index; CA19-9, carbohydrate antigen 19-9; CA12-5, carbohydrate antigen 12-5; CEA, NA = not applicable, HR = hazard ratio; CP, combined phase.

Table S6 Baseline Characteristics of Patients in the biological basis cohort

Variables	Overall (n=15)
Male: Female	10 (66.67%):5 (33.33%)
Age (years)	62.47 ± 5.45
Tumor size (cm)*	3.63 ± 1.13
Operative methods	
Whipple	9 (60.00%)
Others	6 (40.00%)
Rad-score grading	
Low Rad-score	7 (46.67%)
High Rad-score	8 (53.33%)

Note. —Unless indicated otherwise, the data presented are the number of patients, with percentages in parentheses. TNM = TNM Classification of Malignant Tumors.

*Data are means ± SDs, with ranges in parentheses.

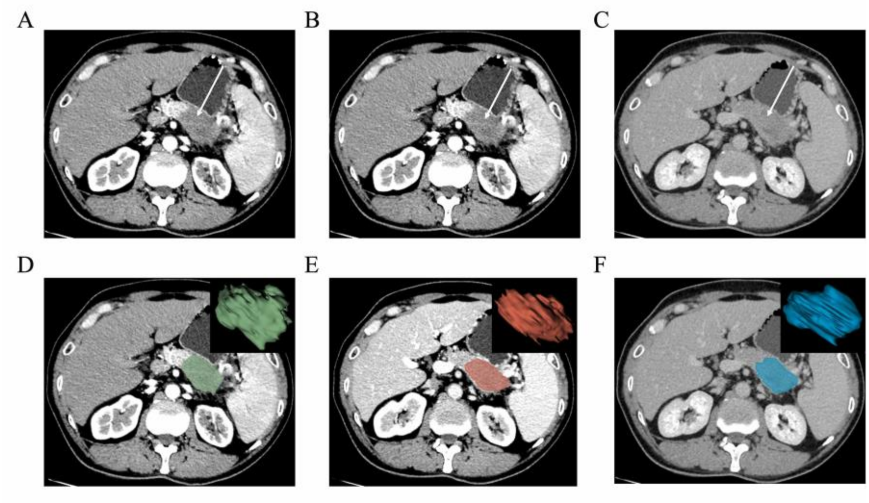

Figure S1: Images in a 57-year-old female with a 32mm PDAC in the tail of the pancreas and a serum CA19-9 level of 347.8 U/mL. Axial CT scans in the (A) AP; (B) PVP and (C) DP show the tumor (white arrow). CT images show the tumor segmentation masks in the (D) AP (green); (E) PVP (red) and (F) DP (blue) and the corresponding volume-rendering images.

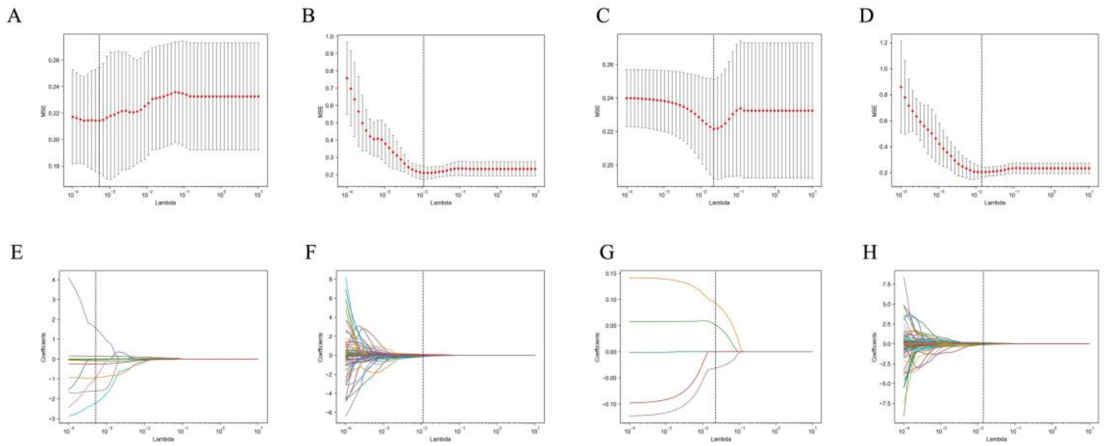

Figure S2: Radiomic features selection using the Lasso logistic regression model. The MSE of different numbers of radiomics features revealed by the Lasso model in AP, PVP, DP, and CP of training cohort. A dotted vertical line is drawn at the optimal lambda values by minimum criteria, which is 1.39×10^{-2}, 5.17×10^{-4}, 1.92×10^{-2} and 2.22×10^{-2} in AP (A), PVP (B), DP (C), and CP (D). The lambda min means the lambda at which the minimal MSE is achieved through five-fold cross-validation. Lasso coefficient profiles of radiomics features. The dotted vertical line was plotted at the optimal λ, resulting in 6, 11, 3 and 12 features with nonzero coefficients for AP (E), PVP(F), DP (G) and CP (H). MSE = mean-square error; AP = arterial phase; PVP = portal venous phase; DP = delayed phase; CP = combined phase.

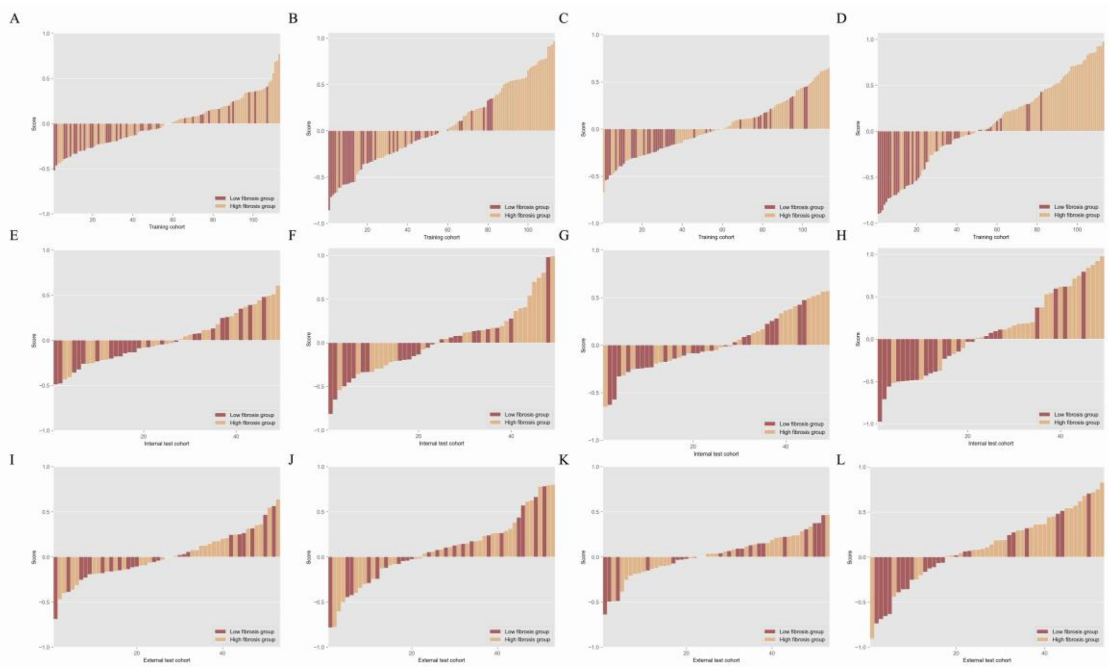

Figure S3: Plots showing the distribution of radiomics model scores for different phases in each dataset. (A)-(D) Training cohort of AP; PVP; DP and CP, (E)-(H)Internal test cohort of AP; PVP; DP and CP, (I)-(L) External test cohort of AP; PVP; DP and CP. The different groups of the fibrosis content are marked with different colors.

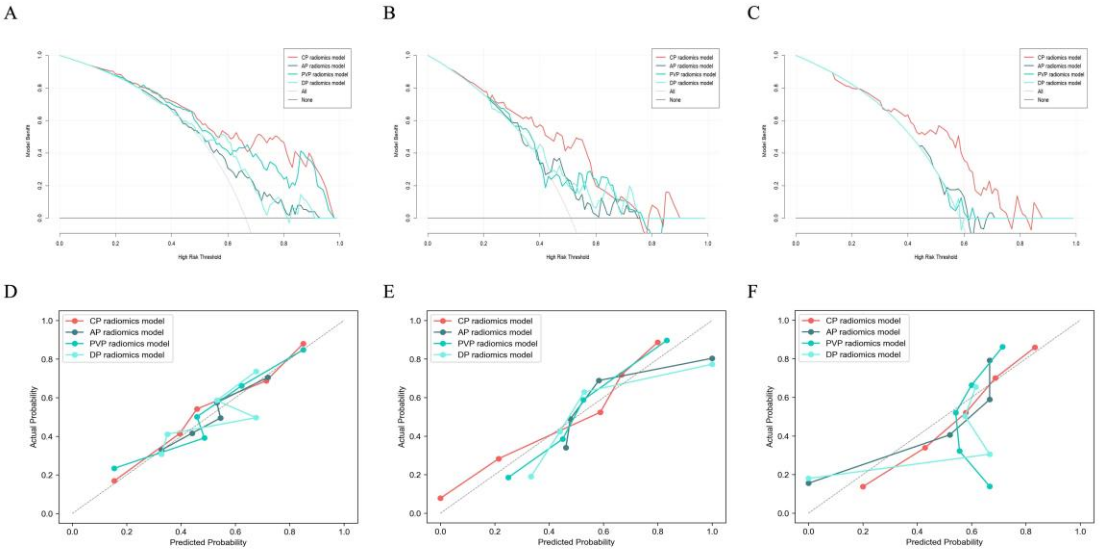

Figure S4: Calibration curves of the model calibration performance of the radiomics model for predicting different fibrosis groups probability in the training cohort (A), internal test cohort (B), and external test cohort (C). Decision curves for different fibrosis groups predictions were obtained by using the established training cohort (D), internal test cohort (E), and external test cohort (F).

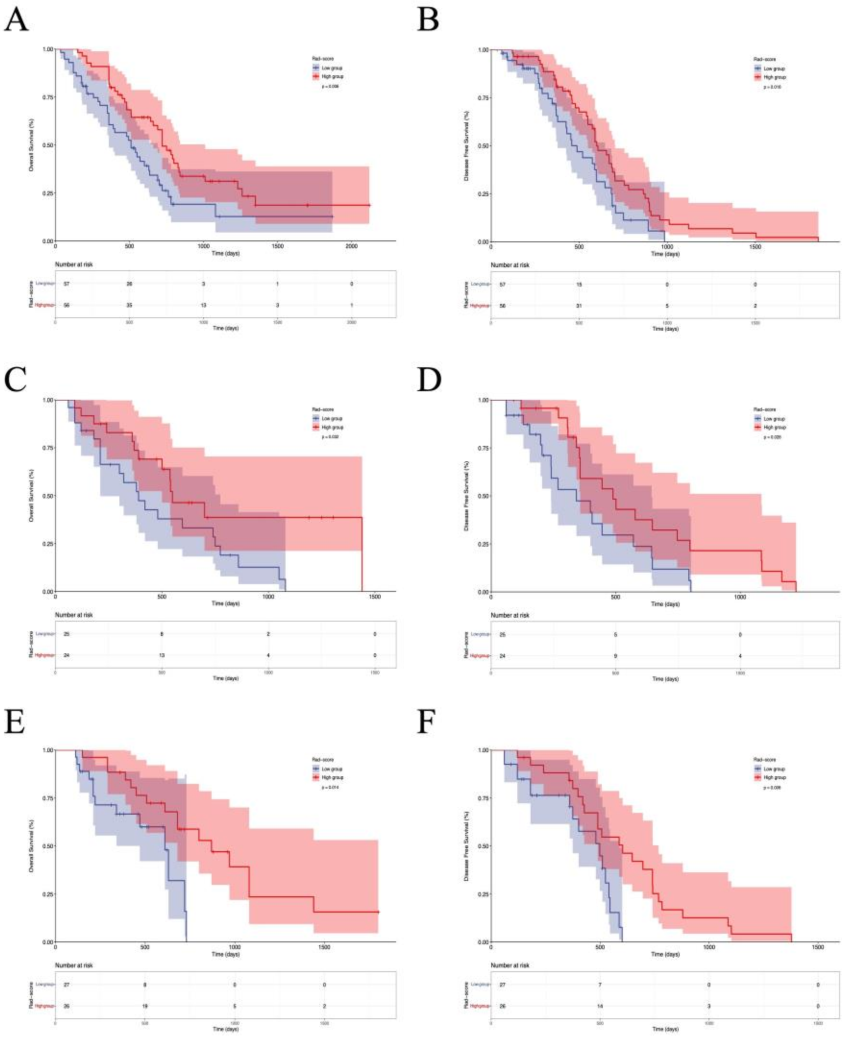

Figure S5: Kaplan-Meier curves of OS and DFS in the training cohort, internal test cohort and external test cohort. The OS and DFS were estimated using Rad-score derived from the CP radiomics model in the training cohort(A)-(B), internal test cohort (C)-(D) and external test cohort (E)-(F) (low or high, as defined by the 0.5 vale of Rad-score).
